# ALK-Brain Prognostic Index—Preliminary Study of a Prognostic Tool for Patients with ALK-Rearranged, Non-small Cell Lung Cancer and Brain Metastases

**DOI:** 10.3390/cancers12071804

**Published:** 2020-07-06

**Authors:** Georgios Tsakonas, Caroline Kamali, Luigi De Petris, Signe Friesland, Rolf Lewensohn, Simon Ekman

**Affiliations:** 1 Theme Cancer, Medical Unit Head & Neck, Lung and Skin Cancer, Karolinska University Hospital, 17176 Stockholm, Sweden; caroline.kamali@ki.se (C.K.); luigi.depetris@ki.se (L.D.P.); signe.friesland@sll.se (S.F.); rolf.lewensohn@ki.se (R.L.); simon.ekman@ki.se (S.E.); 2 Department of Oncology-Pathology, Karolinska Institutet, 17176 Stockholm, Sweden

**Keywords:** ALK translocation, brain metastases, non-small cell lung cancer, prognostic score, overall survival, ALK-Brain Prognostic Index

## Abstract

Background: Disease-specific Graded Prognostic Assessment (DS-GPA) is the most validated prognostic tool for patients with brain metastasized lung cancer. The Lung-molGPA scoring system was recently introduced for oncogenic-driven brain metastasized lung cancer, but has not yet been validated in cohorts including only ALK-translocated tumors. Methods: We designed a retrospective cohort study consisting of 44 patients with brain metastasized ALK-positive, non-small cell lung cancer (NSCLC) who were treated between January 2009 and November 2019 at Karolinska University Hospital in Stockholm, Sweden. Information about demographics and clinicopathological parameters were collected. Predictors of overall survival (OS) were identified by Cox regression analyses. A bootstrap validation with 1000 samples was performed in order to compare the different prognostic scores. Results: The variables found to independently influence OS in the multivariate analysis, i.e., PS, sex and brain metastases at diagnosis, were used as prognostic variables in our new prognostic index (ALK-BPI). Patients were divided into two prognostic groups. The median OS was 65.7 months for the good prognostic group and 22.7 months for the poor prognostic group (*p* = 0.0068). In the univariate analysis of the different prognostic scores, ALK-BPI performed better than the others (HR = 3.6; 95% CI: 1.3–9.9). The mean C-statistics of the different prognostic scores were compared to each other, and no significant difference was observed. Conclusion: We propose the ALK-BPI score as a new prognostic tool that can easily be applied for ALK-positive lung cancer patients with brain metastases in daily clinical practice, as it has at least the same prognostic value as Lung-molGPA.

## 1. Introduction

One of the first oncogenic drivers to be reported in lung cancer was the fusion of echinoderm microtubule-associated protein-like 4 (EML4) and anaplastic lymphoma kinase (ALK) (EML4-ALK) in 2007 [1]. Subsequently, other gene fusion partners to ALK were identified; EML4 is the most common of these, and ALK-rearrangements are found in 4–7% of non-small cell lung cancer (NSCLC) patients, mostly in younger patients with light or nonsmoking history and adenocarcinoma histology [2,3]. Patients with advanced NSCLC harboring ALK rearrangements are routinely treated with ALK tyrosine kinase inhibitors (TKIs), including the first generation crizotinib, the second generation ALK TKIs alectinib, brigatinib and ceritinib, and the third generation lorlatinib [4].

Brain metastases (BM) are among the leading causes of morbidity and mortality in patients with NSCLC [5]. Around 45% of patients with advanced nononcogenic-driven NSCLC and up to 70% of oncogenic-driven NSCLC develop BM over the course of their disease [6,7,8,9]. In newly diagnosed, advanced ALK-positive lung cancer, the incidence of BM varies from 20% to 30%, and the overall incidence of postdiagnosis BM increases over time, standing at 23.8% after 1 year, 45.5% after 2 years and 58.4% after 3 years [10,11].

The prognosis of patients with brain metastatic ALK-rearranged NSCLC has become significantly better thanks to the introduction of targeted therapies [12]. Second-generation TKIs ceritinib and alectinib are approved in the first-line setting, and brigatinib in the second line; their use reduces the risk of developing BM [13,14]. A meta-analysis showed that ALK inhibitors are effective in the brain, irrespective of prior anti-ALK treatment [15].

Several prognostic scoring systems have been used to aid decision making and for estimating survival for patients with lung cancer and BM. Recursive Partitioning Analysis (RPA), a prognostic score developed and used only in patients who receive radiotherapy for brain metastatic disease [16], and Graded Prognostic Assessment (GPA) [17] are the most widely used and validated scoring systems. The initial GPA-score was published in 2008 and thereafter validated in other cohorts [18,19,20]. The variables that proved to be significant in the GPA scoring system were Karnofsky performance status, age, presence of extracranial metastases and number of brain metastases. The disease-specific (DS) GPA, published in 2012, does not differ from the original publication regarding NSCLC patients with BM [21]. The updated Lung-molGPA scoring system was published in 2016, based on 2186 patients diagnosed with NSCLC and BM from 2006 to 2014 [22]. Sperduto et al. incorporated molecular characteristics (EGFR and/or ALK alterations) into previous significant variables, but their work has not yet been validated in large cohorts with only ALK-positive tumors [23]. A recently published Chinese study failed to validate Lung-molGPA as a prognostic score [24].

In the era of targeted therapy, clinically meaningful prognostic scores are important tools that can guide physicians’ decision making and help to inform patients and their families. The aim of our study is to explore the validity of a Lung-molGPA index in an ALK-positive lung cancer cohort with BM, and propose a new prognostic scoring system which can easily be applied in clinical practice. 

## 2. Methods

### 2.1. Patients

We designed a retrospective cohort study. We reviewed 106 patients with advanced ALK- positive NSCLC who were treated between January 2009 and November 2019 at Karolinska University Hospital in Stockholm, Sweden. The cut-off date for data analysis was 8 November 2019. Fifty-four patients were diagnosed with BM and 44 patients received ALK-TKI as first-line therapy after BM diagnosis. The latter group was included in our analyses. We collected demographic data, information about given oncological therapy, histopathology and physicians’ evaluations of performance status before receiving ALK TKI for BM disease (Table 1). Ethical approval was obtained from the regional ethical review board in Stockholm.

### 2.2. Lung-molGPA and DS-GPA 

We divided the patients into Lung-molGPA/DS-GPA groups according to their age at the time of BM diagnosis, KPS, number of BM and presence of extracranial disease (Table 2). In 68% of the patients, a brain MRI with contrast was performed; the remaining patients (32%) had CT-verified metastases. However, all patients had >4 BM, which did not influence the GPA scoring. The presence of extracranial disease was confirmed with a CT scan of the thorax and abdomen in all patients. CT scans of the thorax and abdomen were performed ≤ 4 weeks after the BM diagnosis. Patients were divided into four Lung-molGPA groups; Group 1 (0–1.0 points), Group 2 (1.5–2.0 points), Group 3 (2.5–3.0) and Group 4 (3.5–4.0 points), where Group 1 was the worst prognostic group and Group 4 the best. We also divided patients into four DS-GPA groups; Group 1 (0–1.0 points), Group 2 (1.5–2.5 points), Group 3 (3.0) and Group 4 (3.5–4.0 points), where Group 1 was the worst prognostic group and Group 4 the best. 

### 2.3. Statistical Analysis

Descriptive statistics were performed to analyze categorical and continuous variables. The primary endpoint was overall survival defined from the date of BM diagnosis until the date of the last follow-up or the date of death from any cause. The hazard ratios (HR), together with 95% confidence intervals (CI), were assessed in univariate Cox proportional hazard regression analyses with age, sex, PS, primary vs. secondary BM, presence of extracranial metastases, CNS radiotherapy, number of BM, DS-GPA and Lung-molGPA as independent variables. In a next step, statistically significant independent variables were further included in a Cox regression multivariate model initially excluding the GPA-scores. The independent variables that were found to be statistically significant in the multivariate analysis were used in our new prognostic score. Due to the low number of patients in our cohort, we used the statistical method of bootstrap validation in order to further evaluate the prognostic performance of our model. A bootstrap validation with 1000 samples was performed, and the mean C-statistic over the bootstrap samples was used as a measure of model performance, which was also statistically compared to the prognostic scores. The Kaplan–Meier approach with the log-rank test was then used to compare overall survival between the different groups of the abovementioned prognostic scores. A two-sided *p*-value of <0.05 was considered statistically significant. All analyses were performed using the Statistical Package for Social Sciences (SPSS, IBM, New York, NY, USA) version 25 and R version 3.61.

## 3. Results

### 3.1. Baseline Characteristics

The patients’ clinicopathologic characteristics are summarized in Table 1. The median age at diagnosis was 58 years (range 30–79) with a male predominance (54.5 %). Adenocarcinoma was the dominant histological subtype (95.5%) with only one adeno-squamous carcinoma and one large cell carcinoma. The Eastern Cooperative Oncology Group (ECOG) performance status was < 3 in 77.3% of the cases. Among the group, 61.4% were never smokers and 36.4% were former smokers. All patients received ALK-TKI as a first line treatment after the diagnosis of BM. Fourteen patients had primary brain metastatic disease.

### 3.2. Univariate and Multivariate Analysis

Univariate analysis showed that BM at diagnosis (HR *=* 0.17; 95% CI: 0.05–0.58), PS (HR = 0.32; 95% CI: 0.13–0.79) and sex (HR = 0.40; 95% CI: 0.17–0.95) were prognostic factors for OS. All of these variables remained statistically significant independent prognostic factors in the multivariate OS analysis (Table 3), and were, therefore, included in our prognostic score.

### 3.3. ALK-Brain Prognostic Index (BPI)

**Figure 1:**
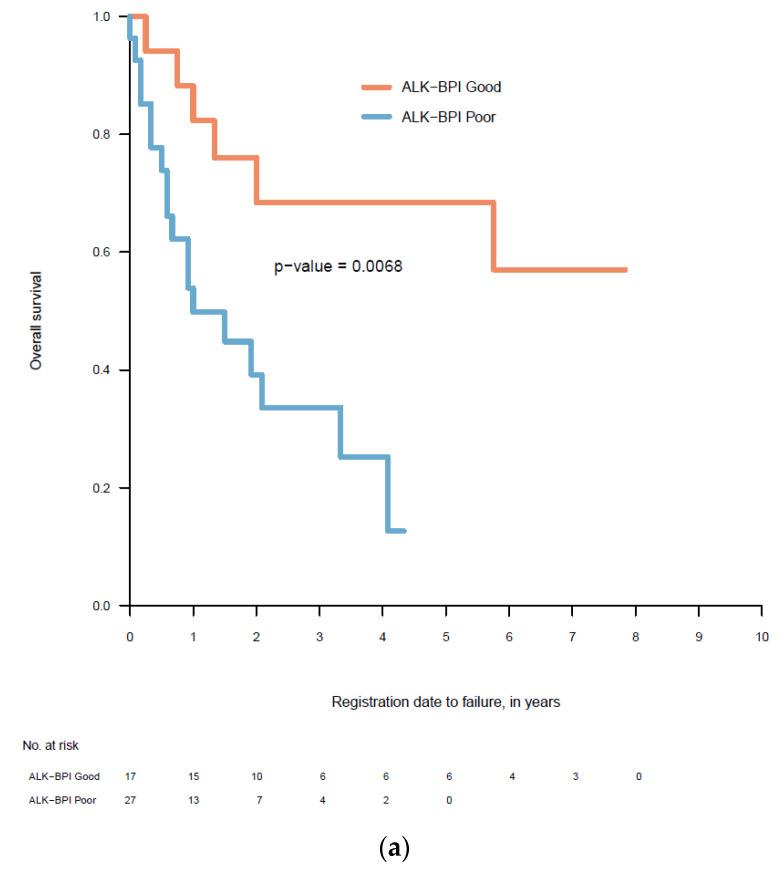

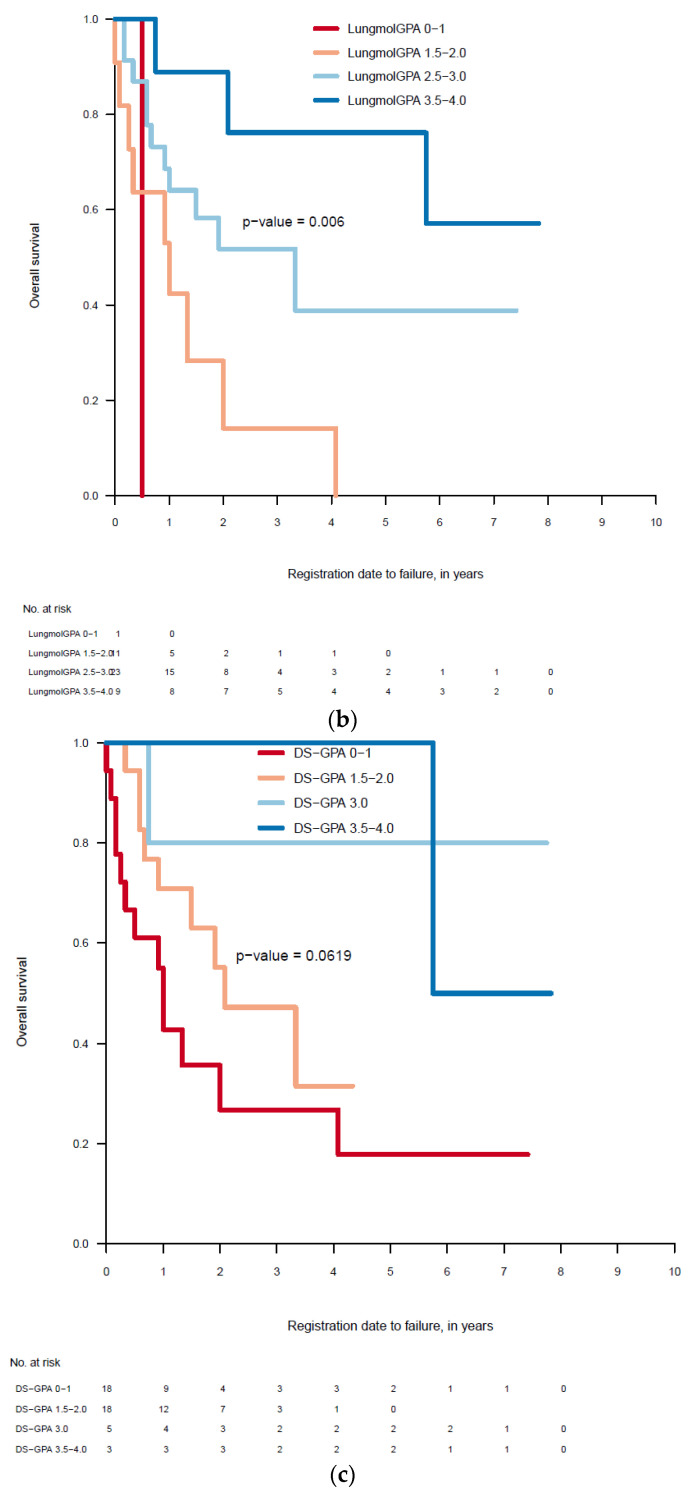
Kaplan Meier curves of the three prognostic scores. (**a**) ALK- BPI: the pairwise log rank test showed a statistically significant difference with *p* = 0.0068 between the good and poor prognosis group; (**b**) Lung-molGPA: a pairwise log rank test showed a statistically significant difference with *p* = 0.006 between all groups; (**c**) DS-GPA: a pairwise log rank test did not show any statistically significant difference (*p* = 0.0619) among any of the prognostic groups.

The variables found to independently influence OS in the multivariate analysis, i.e., PS, sex and BM at diagnosis, were used as prognostic variables in our newly proposed ALK-Brain Prognostic Index (ALK-BPI). A maximum score of 1.0 was assigned to factors with larger effect estimates, such as BM at diagnosis (HR = 0.14; 95% CI: 0.04–0.48), and a maximum score of 0.5 was assigned to smaller effect estimates. The latter included PS < 3 (HR = 0.29; 95% CI: 0.11–0.79) and female sex (HR = 0.27; 95% CI: 0.11–0.67). The ALK-BPI scoring system is presented in Table 2. We divided patients into two prognostic groups. The good prognosis group (1.5–2.5 points) had a median overall survival (mOS) of 65.7 months (95% CI: 47.3–84.1) and the poor prognostic group (0–1.0 points) had a mOS of 22.7 months (95% CI: 14.8–30.5). The Kaplan- Meier curves of the ALK-BPI are presented in Figure 1. 

### 3.4. Comparison between the Different Prognostic Scores

The log rank test in the Kaplan-Meier curves was 0.0068, 0.006 and 0.0619 for the ALK-BPI, Lung-molGPA and DS-GPA, respectively (Figure 1). In the univariate analysis of the different prognostic scores, the DS-GPA prognostic groups did not differ significantly. Not all Lung-mol GPA groups differed significantly. However, there was a clear difference between the two prognostic groups in the ALK-BPI (Table 4).

The mean C-statistic over the 1000 bootstrap samples was 0.6354, 0.6772 and 0.6796 for ALK-BPI, Lung-molGPA and DS-GPA, respectively. These mean C-statistics were compared to each other with the *p*-value, and no significant difference was observed (Table S1).

## 4. Discussion

We propose the ALK-BPI scoring system for patients with ALK-positive lung cancer with BM as a robust and easy-to-use prognostic clinical tool. Accurate prognostic information is essential to help clinicians in their treatment decisions and provide information to patients as well as their families.

Prognostic scores like RPA, GPA and DS-GPA were calculated using patients based on clinical study cohorts, and their external validity can therefore be questioned [16,17,21]. Patient inclusion in the aforementioned trials was done in an era when treatment options were limited for ALK-positive lung cancer patients. The updated Lung-molGPA is the first score to include molecular factors; however, the number of ALK-positive patients included in the statistical analysis was not given, and EGFR-mutation positive patients were calculated together with ALK-positive patients [22]. A major limitation of the Lung-molGPA study is that available treatment options for ALK-positive lung cancer patients were also limited in this cohort (patients diagnosed between 2006 and 2014).

Our cohort included only ALK-positive patients with BM that received ALK-TKI in the first-line (patients diagnosed between 2011 and 2019); most of the patients also received second-generation TKIs [14,25,26,27,28], which is considered the standard treatment today. Second-generation ALK TKIs have better intracranial activity, especially alectinib [12,29] and brigatinib [30], when used in a first-line setting. Ceritinib has also displayed good intracranial activity in patients who had previously received crizotinib in ASCEND-1, ASCEND-2 and ASCEND-3 trials [27,31,32]. The median OS in our cohort was 26 months, compared to 12 months in the cohort used to define the Lung-molGPA. In patients with higher Lung-molGPA scores, i.e., 3.5–4.0, the study by Sperduto et al. displayed a median overall survival of about 47 months, whereas in our cohort, it was 71 months. This difference may be explained by the fact that all patients in our study received ALK-TKI (the majority second- generation TKI) as first-line treatment. No significant difference in OS was observed between the best prognostic Lung-molGPA group and the group with 2.5–3.0 points. This result may indicate that four prognostic groups are too many for ALK-positive patients with BM, and brings into doubt the validity of this scoring system in relation to this group of patients. 

Brain metastases at cancer diagnosis, sex and performance status were found to be independent prognostic factors for OS in the Cox regression analysis in our study, whereas other variables used to define the Lung-molGPA score did not perform well. A recently published Chinese study also showed that the number of BM and the presence of extracranial metastases were not significant prognostic factors for ALK-positive patients with BM, and failed to validate the Lung-molGPA score [24]. The three aforementioned variables, which are used in the ALK-BPI, are easily calculated in a clinical setting without the need for radiological evaluations. This important feature of ALK-BPI, together with the fact that it has only two prognostic groups with a clear difference in OS between them, makes it a more attractive prognostic tool.

The major limitations of our study are its retrospective nature and the relatively small number of patients included. On the other hand, this is the first prognostic score calculated in a homogeneous ALK cohort receiving ALK-TKI as first-line treatment for brain metastatic disease. It can be assumed that the number of ALK-positive patients included in the original Lung-molGPA study is of a similar size as in our study, taking into account the duration for which the patients received treatment, the available molecular diagnostic assessment at that time and the percentage of ALK-positive lung cancer in a cohort of nonsmall cell lung cancer patients. The bootstrap analysis with 1000 samples failed to show any statistically significant difference between the different prognostic scores; however, a validation of our results in a larger clinical cohort is needed. This validation is planned to be performed in the near future.

The real-world nature of our study strengthens the external validity of our prognostic score. The results of our study are concordant with those observed in daily clinical practice, where the number of BM and the presence of extracranial disease do not seem to influence OS when patients are treated with second generation ALK-TKIs. The results of the Kaplan-Meier analyses discriminate among the ALK-BPI groups in a better way compared to the other prognostic scores.

## 5. Conclusions

In conclusion, we propose a new ALK-BPI score as a prognostic tool that can be easily applied for ALK-positive lung cancer patients with BM in daily clinical practice, and that has at least the same, if not better, prognostic value as the Lung-molGPA system. The ALK-BPI score is planned to be validated in a larger cohort consisting of brain metastatic ALK-positive patients receiving ALK-TKI in a first-line setting. 

## Figures and Tables

**Table 1:** Baseline demographics and clinical characteristics.

Characteristics	No of Patients * (*N* = 44)
Median age, years (IQ range)	58 (30–79)
Sex	
Male	24 (54.5%)
Female	20 (45.5%)
Histology	
Adenocarcinoma	42 (95.5%)
Adenosquamous carcinoma	1 (2.3%)
Large cell carcinoma	1 (2.3%)
ECOG performance status	
0–2	34 (77.3%)
3–4	10 (22.7%)
Smoking history	
Never smoker/light smoker	27 (61.4%)
Ex-smoker	16 (36.4%)
Smoker	1 (2.3%)
Brain metastases	
1–4	18 (40.9%)
>4	26 (59.1%)
Brain metastasis at diagnosis	
Yes	14 (31.8%)
No	30 (68.2%)
ALK-TKI first line after diagnosis of brain metastases	
Crizotinib	10 (23%)
Ceritinib	13 (29%)
Alectinib	19 (43%)
Brigatinib	2 (5%)
Extracranial metastases	
Yes	28 (64%)
No	16 (36%)
CNS Radiotherapy	
SRS	11 (25.0%)
WBRT	7 (15.9%)
No RT	26 (59.1%)
Alive	
Yes	24 (55.5%)
No	20 (45.5%)

IQ: interquartile range, ALK: Anaplastic Lymphoma Kinase, TKI: Tyrosine Kinase Inhibitor, CNS: Central Nervous System, SRS: Stereotactic radiosurgery, WBRT: Whole brain radiotherapy, RT: Radiotherapy. * The percentage of patients in each subgroup is shown in brackets, if not otherwise specified.

**Table 2:** ALK-BPI, DS-GPA and Lung-molGPA classifications.

***ALK-BPI*** **Prognostic Factor**	**Scoring Criteria** 0	**** 0.5	**** 1.0
Brain metastases at diagnosis	No		Yes
Performance status	3–4	2	0–1
Sex	Male	Female	
***DS-GPA*** **Prognostic Factor**	**Scoring Criteria** 0	0.5	1.0
Age, y	>60	50–59	<50
KPS	<70	70–80	90–100
Number of BM	>3	2–3	1
ECM	yes		No
***Lung-molGPA*** **Prognostic Factor**	**Scoring Criteria** 0	0.5	1.0
Age, y	≥70	<70	
KPS	<70	70–80	90–100
Number of BM	>4	1–4	
ECM	Yes		No
Gene Status	EGFR neg/unk and ALK neg/unk		EGFR or ALK pos

ECM: Extracranial Metastases, KPS: Karnofsky Performance Status, EGFR: Epidermal Growth Factor receptor, ALK: Anaplastic Lymphoma Kinase, neg: negative, unk: unknown.

**Table 3:** Univariate and multivariate Cox regression analysis for OS.

Univariate Analysis	Hazard Ratio (95% CI)	*p*-Value
Brain metastases (primary vs. secondary)	0.17 (0.05–0.58)	0.005
Sex (female vs. male)	0.40 (0.17–0.95)	0.037
PS (<3 vs. 3–4)	0.32 (0.13–0.79)	0.013
Age *	1.03 (1.0–1.10)	0.093
Extracranial metastases (yes vs. no)	0.49 (0.20–1.20)	0.118
Radiotherapy (yes vs. no)	1.24 (0.53–2.86)	0.620
Brain metastases, No (1–4 vs. >4)	0.69 (0.29–1.62)	0.394
**Multivariate Analysis**		
Brain metastases (primary vs. secondary)	0.14 (0.04–0.48)	0.002
Sex (female vs. male)	0.27 (0.11–0.67)	0.005
PS (<3 vs. 3–4)	0.29 (0.11–0.79)	0.015

CI = Confidence Interval; PS = Performance Status; OS = overall survival. * Age was analysed as a continuous variable.

**Table 4:** Univariate Cox regression OS analysis of DS-GPA, Lung-molGPA and ALK-BPI.

Prognostic Scores	Patients, No. (%)	mOS in Months (95% CI)	Hazard Ratio (95% CI)	*p*-Value
**DS-GPA**				
3.5–4.0	3 (6.8)	81.5 (64.2–98.8)	0.19 (0.02–1.46)	0.111
3.0	5 (11.4)	76.2 (46.7–105.7)	0.15 (0.02–1.16)	0.069
1.5–2.5	18 (40.9)	30.0 (20.6–39.5)	0.56 (0.24–1.32)	0.188
0–1.0	18 (40.9)	26.6 (10.4–42.9)	1	0.107
**Lung-molGPA**				
3.5–4.0	9 (20.5)	71.0 (49.1–92.9)	1	
2.5–3.0	23 (52.3)	44.7 (26.5–62.9)	2.5 (0.7–9.2)	0.167
1.5–2.0	11 (25.0)	15.7 (5.0–26.5)	6.5 (1.7–25.6)	0.007
0–1.0	1 (2.3)	6.0 (6.0–6.0)	17.2 (1.6–189.2)	0.020
**ALK-BPI**				
1.5–2.5	17 (38.6)	65.7 (47.3–84.1)	1	
0–1.0	27 (61.4)	22.7 (14.8–30.5)	3.6 (1.3–9.9)	0.012

mOS: median Overall Survival, No: Number, CI: Confidence Interval.

## Supplementary Materials

The following are available online at https://www.mdpi.com/2072-6694/12/7/1804/s1, Table S1: Bootstrap validation of ALK-BPI, Lung-molGPA and DS-GPA.
