# Supplementary material for: ALK-Brain Prognostic Index—Preliminary Study of a Prognostic Tool for Patients with ALK-Rearranged, Non-small Cell Lung Cancer and Brain Metastases
Source: Cancers (Basel). 2020 Jul 6;12(7):1804. doi: 10.3390/cancers12071804 (PMC7408161; doi:10.3390/cancers12071804)
Supplement: Supplementary file 1 [file cancers-12-01804-s001.pdf]

cancers

MDPI

Supplementary Materials

ALK-Brain Prognostic Index—Preliminary Study of a Prognostic Tool for Patients with ALK-Rearranged, Nonsmall Cell Lung Cancer and Brain Metastases

Table S1. Bootstrap validation of ALK-BPI, Lung-molGPA and DS-GPA.

	C-statistics	*p*-Value		
		ALK-BPI	Lung-molGPA	DS-GPA
ALK-BPI	0.6354	-	0.506	0.490
Lung-molGPA	0.6772	-	-	0.925
DS-GPA	0.6796	-	-	-

A bootstrap validation with 1000 samples was performed and the mean C-statistic over the bootstrap samples was used as a measure of model performance. C-statistics between scores were compared with the *p*-value.
